# Cognitive distortions among older adult gamblers in an Asian context

**DOI:** 10.1371/journal.pone.0178036

**Published:** 2017-05-18

**Authors:** Mythily Subramaniam, Siow Ann Chong, Colette Browning, Shane Thomas

**Affiliations:** 1 Research Division, Institute of Mental Health, Singapore, Singapore; 2 Royal District Nursing Service Limited, Melbourne, Australia; 3 International Primary Health Care Research Institute, Shenzhen, China; 4 Monash University, Melbourne, Australia; Universidad de Granada, SPAIN

## Abstract

**Aims:**

The study aims to describe the construct of cognitive distortions based on the narratives of older adult gamblers (aged 60 years and above) in Singapore.

**Methods:**

Singapore residents (citizens or permanent residents) aged 60 years and above, who were current or past regular gamblers were included in the study. Participants were recruited using a combination of venue based approach, referrals from service providers as well as by snowball sampling. In all, 25 in-depth interviews were conducted with older adult gamblers. The six-step thematic network analysis methodology was adopted for data analysis.

**Results:**

The mean age of the participants was 66.2 years. The majority were male (n = 18), of Chinese ethnicity (n = 16), with a mean age of gambling initiation at 24.5 years. Among older adult gamblers, cognitive distortions emerged as a significant global theme comprising three organizing themes–illusion of control, probability control and interpretive control. The organizing themes comprised nine basic themes: perception of gambling as a skill, near miss, concept of luck, superstitious beliefs, entrapment, gambler’s fallacy, chasing wins, chasing losses, and beliefs that wins are more than losses.

**Conclusions:**

Cognitive distortions were endorsed by all gamblers in the current study and were shown to play a role in both maintaining and escalating the gambling behaviour. While the surface characteristics of the distortions had a culture-specific appearance, the deeper characteristics of the distortions may in fact be more universal than previously thought. Future research must include longitudinal studies to understand causal relationships between cognitive distortions and gambling as well as the role of culture-specific distortions both in the maintenance and treatment of the disorder.

## Introduction

Worldwide, older adults constitute an increasing proportion of the population [1]. These older adults are living longer and ageing actively as they continue to participate in a number of activities including gambling. It has been shown that gambling is a popular social activity among older adults across many cultures, [2,3] with a recent systematic review establishing the prevalence of lifetime ‘problem or pathological’ gambling among older adults as ranging from 0.01% to 10.6% [4]. A number of ecological factors (e.g., availability of gambling venues, advertised incentives) and individual level factors (e.g., personal and role losses) may render older adults more vulnerable to gambling problems. Social environment, shared values and cultural norms influence both the decision to gamble as well as the extent of gambling involvement [5]. However, research concerning older adults’ motivation to gamble has been limited.

The cognitive theory remains one of the dominant approaches that explains gambling behaviour [6]. The cognitive theory of gambling posits that gamblers continue to play because they possess distorted beliefs about gambling [7,8] which are often referred to as cognitive distortions or cognitive biases [8–11]. Ladouceur [12] proposed three broad categories of cognitive distortions: (i) gambler’s fallacy, i.e., not accepting the randomness of an event and harboring misperceptions that a future win or loss is related to past outcomes when, in fact, each gambling event is distinct. Thus, gamblers continue to gamble despite a series of losses in the belief that a win will follow losses [7,13]; (ii) illusion of control whereby gamblers attempt to predict or control events that are inherently random thus they incorrectly attribute the result of winning a game to their own actions, which motivates them to develop their skills to increase their winnings [14] and (iii) superstitions—beliefs that certain behaviors, rituals (e.g., wearing a certain coloured shirt or a lucky charm) or thoughts will influence the outcome of a gambling event [14]. Other ccommon cognitive distortions include attribution bias in assigning credit to one’s own skills in winning, blaming external influence for losses, hindsight bias and chasing wins and/or losses [8].

Evidence suggests that cognitive distortions related to gambling activities play a role in the development and maintenance of gambling disorder [12,15]. Compared to non-problem gamblers, problem and pathological gamblers reported greater cognitive biases along the luck/perseverance and illusion of control dimensions [16,17]. Gambling cognitions were found to moderate the relationship between risky gambling practices and gambling intensity [18], and symptoms of pathological gambling remained significantly associated with cognitive distortions after controlling for genetic and shared environmental influence [11].

Loo et al. [19] in their review of culture and gambling have proposed that culture plays a role in both gambling initiation and maintenance, and thus certain cultural groups are more likely to gamble than others. It follows that cultural beliefs may contribute to cognitive distortions and thus affect gambling behaviour. Zitzow [20], in his research on adolescent American Indians and gambling concluded that cultural acceptance of magical thinking and beliefs in luck and fate allows adolescents to generalize the same to gambling, which could be contributory to the higher prevalence of gambling seen among them. Papineau [21] similarly proposed that the Chinese have distinctive notions of fate, luck, risks and control; and hence, the patterns of Chinese gambling are a reflection of their cultural views and traditional beliefs in luck, fate and destiny. Loo et al. [19] concluded that the cultural differences between Chinese and Western gamblers are best explained by familial influences as well as other distorted beliefs and cognitions that are prevalent among Chinese gamblers.

Singapore is a highly urbanized city-nation situated at the southern tip of the Malayan Peninsula. In 2015, the population of Singapore was 5.5 million of which 3.9 million were Singapore citizens and permanent residents [22]. Singapore is diverse, with a multi ethnic population comprising Chinese (74.3%), Malays (13.3%), Indians (9.1%) and ‘Other’ ethnic groups (3.2%). Given the influence of customs and traditions on gambling [19] these ethnic groups differ in terms of their gambling activities. Gambling is a socially accepted and popular activity among the Chinese and often occurs during festive seasons such as the Chinese New Year. Gambling is, however, forbidden according to the tenets of Islam which is the predominant religion of the Malay population in Singapore, thus gambling participation and gambling disorder is significantly lower among the Malays as compared to those of Chinese ethnicity [23,24]. Few studies have focused on gambling trends among the Indians, and not much is known about their gambling behavior, though a previous study has shown that prevalence of pathological gambling is lower among Indians as compared to Chinese [23]. Studies have also shown that gambling is a popular activity among older adults in Singapore with the lifetime participation rate (of gambling) among older adults reported as 56.2% and 48.9% in two recent studies [25,26]. While Tse et al. [25] reported that 2.2% of older adults were problem gamblers; a study by National Council of Problem Gambling, Singapore [24] reported a slightly higher prevalence of pathological gambling among the older age group. However, very little research has been done among older adults of this multi-ethnic population that provides an explanatory framework of their gambling.

The study used data from a larger qualitative study exploring gambling initiation, maintenance, harm, help-seeking and barriers to care among older adults in Singapore, where older adults were defined based on the United Nation’s definition of those aged 60+ years as older persons [27]. A qualitative methodology was adopted given both the lack of previous studies in this population and to understand the phenomenon from the perspectives of the older adult gamblers. Cognitive distortions emerged as a salient theme in the maintenance of gambling spontaneously and consistently in the narratives of older adult gamblers. The current study thus aims to describe the construct of cognitive distortions based on the narratives of older adult gamblers in Singapore.

## Materials and methods

### Research participants

Inclusion criteria for the study included Singapore residents (citizens or permanent residents) aged 60 years and above, who could speak English, Chinese, Malay or Tamil and were current or past regular gamblers (defined as those who reported gambling at least weekly beyond the age of 60 years). Participants were recruited using a combination of network and purposive sampling. Recruitment was carried out in gambling venues by approaching participants directly based on appearance (i.e., looked like older adults). The study was then briefly described to them and they were given leaflets that provided details of the study as well as the contact information of the first author (MS) and were asked to contact the researcher if they met criteria for the study. The other source of recruitment was a tertiary treatment centre for addictions in Singapore; counselors and clinicians providing treatment to those with gambling disorder [28] (as diagnosed based on the Diagnostic and Statistical Manual of Mental Disorders, 4th. Edition (DSM-IV- TR) criteria) referred suitable clients to the researcher. A snowball sampling approach was also used by requesting participants to refer anyone they knew who met the inclusion criteria for the study. Efforts were made to ensure sample diversity in terms of gender, ethnicity and severity of gambling problems by refining the sampling when needed. Referring clinicians were actively engaged and asked to refer participants with specific socio-demographic characteristics. Women and those of Malay and Indian ethnicity were given priority during the recruitment process as there were fewer older adult gamblers in these subgroups.

Sample size was guided by the concepts of “theoretical saturation” [29] and “redundancy” [30]. Along with ongoing analysis to identify new information and emerging themes, a detailed analysis of data was conducted after the 10^th^, and the 25^th^ interview. The analysis after the 25th interview was discussed amongst the researchers and the subsequent consensus was that ‘theoretical saturation’ had been achieved.

### Study sample

In-depth interviews were conducted with 25 older adults (60 years and above) who were current or past regular gamblers. A simple screening was done over the telephone to ensure that the participant met the inclusion criteria and an appointment was scheduled for face-to-face interview. All interviews were conducted by the first author (MS) who is trained in qualitative methodology.

The study was given ethical approval by the National Healthcare Group Domain Specific Review Board, Singapore. After obtaining written informed consent, each respondent was interviewed using an interview guide (S1 File). Each interview varied between 45 minutes to 2 hours in duration and was held at a venue preferred by the respondents. All the interviews were conducted in English and were audio recorded which were subsequently transcribed verbatim.

Socio-demographic information on age, age of onset of gambling, gender, ethnicity, marital status, education and employment was also collected. The South-Oaks Gambling Screen (SOGS) [31] was used to confirm the life-time diagnosis of probable pathological and problem gambling in the participants. SOGS has been validated in the Singapore population [32]. Respondents scoring 5 or more on the SOGS were categorized as ‘probable pathological gamblers’, those scoring 1 to 4 were categorized as ‘problem gamblers’ and those scoring 0 were categorized as ‘non-problem gamblers’.

The socio-demographic characteristics of the sample are shown in Table 1, and the types of gambling activities of the older adult gamblers in the sample are shown in Table 2. The mean age of the participants was 66.2 years. The mean age of gambling initiation was 24.5 years (standard deviation (SD) = 10.6 years, range 9 to 50 years), while the mean duration of gambling was 41.1 years (SD = 13.4 years, range 10 to 65 years). Nine respondents met lifetime criteria for probable pathological gambling using the SOGS criteria.

**Table 1:** Characteristics of 25 older adult gamblers.

Socio-demographic Characteristics	n
**Age at time of interview**	
*Range*	60–81 years
*Mean (SD)*	66.2 (6.5) years
**Ethnicity**	
*Chinese*	16
*Malay*	2
*Indian*	4
*Others*	3
**Highest Education Attained**	
*Primary*	2
*Secondary*	9
*A Level*	3
*Diploma*	3
*University Degree and above*	6
*Others*	2
**Marital Status**	
*Single*	2
*Married*	20
*Divorced/Separated*	1
*Widowed*	2
**Employment**	
*Employed*	2
*Unemployed*	15
*Retired*	6

**Table 2:** Lifetime gambling activities among the older adult gamblers.

*Gambling Activity (positive endorsement)	
Played cards for money	16
Bet on dogs/horses or other animals	12
Bet on sports	6
Went to casinos	14
Played the numbers or bet on lotteries	22
Played bingo	8
Played the stock or commodities market	9
Played the slot machine/poker machine	12
Bowled/shoot pool/ played any game of skill for money	6

*Most of the older adult gamblers had participated in more than one form of gambling

### Data analysis

The six-step thematic network analysis methodology proposed by Stirling [33] was adopted. It involves identification of basic themes’ that represent the lowest order premises and are grounded in participant experience. These themes are compared and contrasted for similarities and differences and then grouped into more abstract ‘organising themes’ guided by the researcher’s interpretations. Organizing themes are then arranged into a higher order, global theme that encompasses a major point in the text [33]. These basic, organizing, and global themes can be displayed, visually, as a network. The fourth stage involved describing and exploring the networks generated, which are then summarized and finally, the key conceptual findings in the summaries of each thematic network are woven together and used to answer the original research questions.

The first author (MS) identified codes in the text by familiarization with the data through active reading and re-reading of all the interview transcripts. A detailed analysis was conducted following Stirling’s methodology using NVivo software. Another researcher (ST) independently coded the first 5 transcripts and codes and themes identified were discussed and refined. All the coding was done subsequently by MS. Codes and themes were also discussed with two other researchers (ST and SAC) throughout the analysis.

## Results

**Fig 1:**
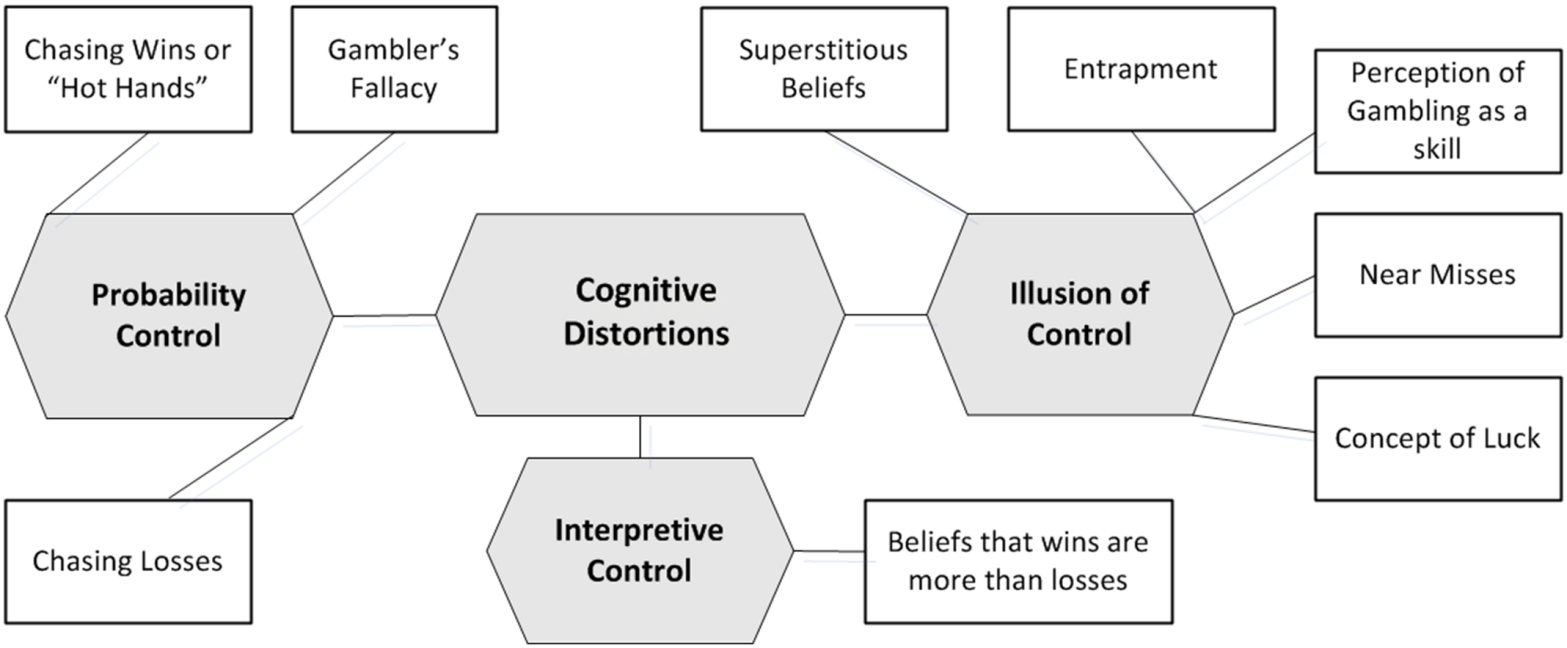
Thematic network representing cognitive distortion among older adult gamblers.

Among older adult gamblers, cognitive distortions emerged as a significant global theme comprising three organizing themes–illusion of control, probability control and interpretive control. The organizing themes comprised nine basic themes: perception of gambling as a skill, near miss, concept of luck, superstitious beliefs, entrapment, gambler’s fallacy, chasing wins, chasing losses, and beliefs that wins are more than losses. The organizing and basic themes were named based both on previous research [8,12,15] as well as the authors input based on their domain knowledge. The three organizing and nine basic themes are described in further detail below (Fig 1).

### Illusion of control

This included beliefs that one could either actively exert control over gambling outcomes either by their own skills or through the use of external forces which included reliance on use of superstitions and rituals or passively by believing in luck.

#### Perception of gambling as a skill

Older adult gamblers (n = 14) saw gambling as a game involving skill. They perceived games like sports betting, race horse gambling and Mahjong as requiring intelligence, skills, analysis, and practice. They felt that by spending time and developing their skills they could get better in these games. They often talked about the superiority of their skills and their mastery of the game. Surprisingly, some lottery players also perceived lottery as a game of skill and one that requires studies of past wins and use of complex analysis to generate the right number.

*“After sometime you pick up. Then what I like about horse racing is that you have to study. So, it’s not just the game…not a game of luck, like casino”*.

(PG 1001)

*“I spent a lot of time on the track in the Turf club. I like horses, I see them run, I see them train. The trainers are all like my friend. From there I learn about judging a horse. I can see a superior horse and I can choose a winner”*.

(PG1022)

*I try to make a chart and see what I can get out of it. I see and log in all the numbers every week that appear (in the newspapers as winning numbers), then I start to study from there, which numbers are always winning and which are not. I pick those numbers and I try to get about, 15 numbers out of it, then I roll it (change the order of the numbers)….and I win regularly*.

(PG1020)

#### Near miss

Near-misses were reported by some gamblers (n = 4) and often perceived as a reason for wanting to continue gambling. This was stated in relation to slot machines when they would get two matching icons on the payline, with the third match stopping just above or below the payline. It was also reported by lottery gamblers who reported missing out on the top prize by just one digit or that the winning number was a permutation of their personal favorite number.

#### Concept of luck

Older adult gamblers ascribed their winning or losing over the years to “luck”. Many (n = 16) felt that they were lucky and that they had always won something. They also talked about a lucky period in their lives when they won consistently or days on which they were so lucky that they won every game. They felt that luck determined winners and mentioned the big lottery or casino winners who had been highlighted in the media and attributed these big wins to luck rather than the exercise of gambling skills. They also felt that some people were ‘unlucky’ and associating with them could lead to being tainted with bad luck. Respondents also talked about specific lottery outlets or casinos that they considered to be lucky. These were those associated with personal wins or those which the media had featured as the outlets where the prize winning lottery ticket was sold.

*“I have got this ladies luck. But people tell me you got ‘lady luck’ first time come you win. Just choose the number you like, buy 5 dollar one ticket. I will win. Something like that… But every time I have this luck*.

(PG1004)

*“And that’s luck. You know, I mean you can be skillful, it’s just … I would say about 25% is skill, observation, you know, through your memory but really 75% luck”*.

(PG1008)

#### Superstitious beliefs

Most of the older adult gamblers (n = 18) believed that carrying out certain rituals or behaviours would tip the gambling outcomes in their favour. One of the older adult gamblers admitted that he would make it a point to wear clothes of specific colors whenever he gambled. Others talked about believing in the luck of colors especially when they had not been winning for some time. Lottery gamblers often mentioned ‘hot numbers’; these numbers were often associated with the death of a well-known person or a catastrophic event. The date and year permutation/combination on which the event happened were considered “hot numbers”. They would talk anecdotally of big wins in the past associated with such incidents and also of such numbers selling out quickly in lottery outlets. Any bad event was also paradoxically associated with good luck, e.g., if a car was involved in an accident or a person was hospitalized–the car number or bed number (in the hospital) was then considered to have high probability of being the winning lottery number. They felt that if something bad or unfortunate was associated with a number, something good must follow from it. Some lottery gamblers purchased lottery numbers that had special meaning to them such as the numbers associated with the address of their first house or the license number of their first car as they felt these were significant and lucky events for them. Some of the lottery gamblers mentioned ‘seeing’ the numbers in a dream while others associated a specific scene such as sky or fire (seen in their dreams) with specific numbers, while others used complex analysis to derive their lucky numbers. They also mentioned that certain numbers ‘appeared to them’, i.e., they saw the same number repeatedly on license plates of cars, in media reports and as house numbers and felt that these numbers were lucky and must be bet upon.

*“Those other days I have very special number. I can even dream … dream the winning number in 4-D (local form of lottery) that is why I strike big 14K, 17K, 35K. I can still remember the date until now. You can check”*.

(PG 1004)

*“You must dream about these numbers and they knew the connections between the dreams and numbers, if you dream about this it means this number, you dream about that it is that number….. If you see someone that you have not seen for a long time it is connected to a number”*.

*(PG1017)*.

Some of the superstitions endorsed by older adult gamblers seemed unique to the Asian culture, e.g., lottery gamblers had a penchant for trying to discern numbers in the markings found on the body of a particular species of fish which is called the “Luo Han” in the local Chinese vernacular. (A distinctive trait of this fish is its flowery black markings on both sides of its body. These side markings are said to variously resemble lottery numbers)

*“Before- I rear fish … the—Luohan fish, the body got number. I see the body, the skin; the body ah got the number, that’s why I buy that number- 3266”*.

(PG1005)

Older adult gamblers prayed to certain Gods or carried out certain specific rituals such as taking a bath with water scented with certain flowers that are considered to have the powers to bestow them with luck. Indian gamblers spoke about identifying ‘a good time’ of the day using traditional almanacs and buying lottery tickets during the identified window period, while Chinese gamblers would avoid entering gambling venues through the main door but would enter by the side door or back door instead.

*“See, on Chinese New Year, I have got a plant in my house, that plant bloomed, bloom flowers. And we say that the plant bloom flowers, it got luck. So, I was thinking, ‘Oh, my luck is coming lah.’ So, this is one of the triggers. See, my luck is coming. So I better go and buy. So that tempts me to go and buy, buy 4D, you see”*.

(PG 1002)

*“Last time I can gamble until, I go and ask the God “eh today can go casino you know or not? Then you throw the coin, then they will answer you: “yes” then you go”*.

(PG 1016)

#### Entrapment

Another basic theme that emerged especially pertaining to lottery gamblers was the profound reluctance and fear to stop betting on a particular number. Older gamblers (n = 8) who had bet on a specific number over many years and expressed feelings of anxiety if they were unable to purchase that number for the draw on that day as they worried that the number would strike big when they did not buy it. They felt compelled to buy the number and had a sense of fear when they thought of ‘letting the number go’.

*“I find it very difficult stop because every Wednesday, Saturday and Sunday around 5’o clock, I must go and line up to buy 4D. I don’t know what it is…there is urge, there is strong urge who ask me to go and buy. Don’t buy your favorite number will come out”*.

(PG 1002)

“I play a number so long. As soon as I drop the number … [expletive]… the number comes out seriously, sorry for my language. So there are some people who are gambling, who are playing with the same number for the last three years and never came out you see, and they are so afraid to like let it go.”

(PG1020)

### Probability control

Older adult gamblers often assumed that events were related when they were not, and viewed independent outcomes as dependent outcomes. Distortions such as these included gambler’s fallacy, “hot hands” and chasing losses.

#### Gambler’s fallacy

Consistent with the fallacy, some of the older adult gamblers (n = 5) felt that while their luck was not good currently, it was bound to change in the future. They felt that if one continued to play long enough one would start winning. Lottery gamblers who bet on a specific number were convinced that since the number had not ‘struck big’ for a long time, it would be a matter of time before this number would be the winning one.

*“No, no, it’s automatically. The …the devil behind my back, you know, he was telling me, your luck is coming. Give another try. Hmm…your luck is coming. Don’t miss the opportunity. See…you have the thinking that your luck is coming”*.

(PG1002)

#### Chasing wins or ‘hot hands’

Gamblers also believed in lucky streaks; some of them (n = 4) felt that if they had a win, they will continue to win up to three times and this often led them to gamble more heavily or for a much longer time after a win. Other older gamblers mentioned betting on numbers that had a history of wins.

*“Sometimes I go off a bit, you now, because of the greed, feeling that I am sure going to win this time- sure going to win lah but then nothing happens”*.

(PG1020)

#### Chasing losses

Older adult gamblers often reported chasing losses (n = 11), i.e., continuing their betting or increasing the amount of their bets in order to recover their monetary loss. Respondents reported betting heavily and playing for longer hours in an attempt to recoup their losses; chasing was also associated with irrational beliefs such as believing that their luck was bound to change with subsequent bets and changing slot machines would also change their luck.

“And the worst thing that can happen, you keep chasing after horses, when you are down. One of my biggest losses was on a horse. Was about 28 thousand”

(PG1001)

*“And as you know, when you are losing, you start losing your ability to think straight. Just like horse racing, like now, I come to a stage whereby I say, "I lost that big amount because I got desperate and instead of saying, 'Okay, that's it. I lose this amount—quit.‴ I try and recover that, and that's where you die”*.

(PG1024)

### Interpretive control

#### Belief that wins are more than losses

Older adult gamblers remembered their wins vividly while often underplaying their losses. They often forgot their losses and convinced themselves that they were not losing money in the long run. Some of them (n = 6) felt that overall they had won more than what they had lost. Many remembered vivid details about these wins such as the venue where it had occurred, the year, name of the horse etc. On the other hand, accounts of losses were not very specific (and vague) with many older adult gamblers emphasising that they often lost what they had previously won and hence it could not be considered a loss. Lottery gamblers emphasised that the amount of money staked was miniscule in comparison to that of the top prize and hence the risk was worth it.

“But I didn’t lose lah. From that time I take every one year once or two years once I will strike 5,000, 10,000 like that so why not… try?”

(PG1013)

“No, loss no, because I never gamble heavily. I gamble, at the most, one week is about $60, $70. And I've struck 18K, 16K to 12K. So for three times I made some money, first prize. Then Toto (a local form of lottery) I make—every now and then, you don't get much, but $90, $60—it is quite common.”

(PG1015)

The endorsement of each of the basic themes by the older gamblers is shown in Table 3.

**Table 3:** Endorsement of each basic theme by older adult gamblers.

Endorsement of themes	Total (25)
1. Perception of gambling as a skill	14
2. Near Miss	4
3. Concept of luck	16
4. Superstitious beliefs	18
5. Entrapment	8
6. Gambler’s fallacy	5
7. Chasing losses	11
8. Chasing wins	4
9. Beliefs that wins are more than losses	6

## Discussion

Cognitive distortions endorsed by older adult gamblers from a multi-ethnic Asian background of illusion of control, probability control and interpretive control corresponded with the findings of Toneatto et al. [8]. Illusion of control is defined as the tendency to believe that one’s behavior is the cause of the occurrence of desired outcome, an outcome which in fact occurs independently of any action on the person’s part [34,35]. This distortion might result from attempts to gain control by gamblers in an uncertain environment wherein chances of success are low. Illusion of control is often associated with disordered gambling as gamblers feel that they can influence the outcome of their bets resulting in heavier bets and losses [36,37]. Based on their data, Toneatto et al. [8] classified illusion of control as ‘active and passive illusory control’ which corresponded with using ‘superstitious beliefs and rituals’, ‘perception of gambling as a skill’, and, believing in the ‘concept of luck’ respectively in the current study.

In the current study, some gamblers felt that they could control the outcome of the game using their skills; this belief extended to games of both skill and chance. Gamblers spent considerable time, effort and resources in developing such skills which further strengthened their illusory control and justified their playing. Clark [6] in his review concluded that the illusion of control leads a gambler to perceive a game of chance as one requiring skills, and that in games where there is an element of skill involved the illusion leads gamblers to believe that the skill is significantly and disproportionately influential. Cantinotti et al. [38] similarly established in their study that higher knowledge about the gambling activity led gamblers to believe that they could predict the outcome of the game. Ohtsuka and Chan [39] have suggested that older Chinese adults play more traditional Chinese gambling games such as mahjong that do require skills and thus tend to believe that their skills influence the outcome of gambling significantly. Zhou et al. [40] suggest that the belief in luck or skills are domain-specific, and vary with the type of gambling activity. Depending on the complexity or difficulty associated with playing a game, gamblers may believe that skills/ luck play a more significant role. Thus while skills may be seen as playing a more significant role in wins in games like mahjong that are perceived as requiring strategy and decision making, lottery gambling may be perceived as more luck based by the majority of gamblers.

Another theme that was endorsed by older adult gamblers was that of near-miss. They felt encouraged by near-misses and perceived them as a sign that they were close to winning which lead to a persistence of gambling. A study by Billieux et al. [41] demonstrated that near misses were associated with higher endorsement of wanting to continue to play and near miss outcomes were aligned with skill oriented cognitions. Reid [42] suggested that while near-miss is not an indication of improving skills in games of chance like slot machines and lotteries, gamblers take them as an indication of their improving skills leading to maintenance of gambling.

Some gamblers considered themselves to be inherently lucky, i.e., they perceived luck as a trait which helped them to win. In contrast to this passive form of control, other gamblers endorsed the use of superstitions that helped them to win. A number of studies have shown that gambling is significantly associated with superstitious beliefs [43–45]. Superstitious beliefs among gamblers include talismanic superstitions–the belief that certain objects and numbers are lucky, behavioral superstitions where certain acts or rituals are believed to be lucky and cognitive superstitions that include prayers and maintaining positive attitudes. This sample of Asian gamblers endorsed all three forms of superstitions some of which were strongly influenced by and unique to this culture such as using Luo Han fish as a means to strike the winning number and association of unfortunate events with lucky numbers. Ohtsuka and Ohtsuka [46] in their work with Vietnamese- Australian gambler had similarly identified culture specific schema–a knowledge structure that is rooted in culture and concluded that such culture-specific beliefs may further strengthen the illusion of control.

Cognitive entrapment is a distortion whereby individuals ‘escalate their commitment to a previously chosen, though failing, course of action in order to justify or make good on prior investments’ [47]. Gamblers in the current study talked about feeling compelled to continue betting on a specific number fearing that if they stopped the number would be drawn thus resulting in profound regret. This pattern of superstitious thinking often led to regular gambling despite mounting losses. Cognitive entrapment has similarly been described by Brockner and Rubin [47] among lottery players, especially when players selected the same numbers each week.

Probability control comprised gambler’s fallacy, chasing wins and chasing losses in the current study. Gambler’s fallacy is defined as a belief that a successful outcome is due after a run of bad luck [48], while the “hot hand” fallacy refers to the belief that if an individual has won in the past (“hot”), they are likely to win in the future. Older adult gamblers often expressed the belief that while their luck might be bad at present, this would change for the better if they persisted in gambling and were certain that after some time they would start winning. This often led to their chasing of losses. On the other hand they believed in ‘lucky streaks’ tending to bet more heavily when they were winning and described buying lottery numbers which had a past history of winning.

Older adult gamblers in the current study tended to recall their wins especially large ones very clearly and vividly, while losses were recalled less easily and vaguely. Wardle et al. [49] also found that gamblers overestimated their winnings and underestimated losses and suggested this may partly be due to the fact that large wins are rare and become especially salient as compared to losses that are frequent and small.

Cognitive distortions were endorsed by all gamblers in the current study and were shown to play a role in both maintaining and escalating the behaviour. Quantitative studies have similarly found that gambling distortions are prevalent even in non-gamblers, but scores increase with gambling involvement, and pathological gamblers have very high scores [50].

The study must be viewed in the light of certain limitations. All the interviews were conducted by the first author (MS) who is proficient in English and Tamil. While research assistants who were proficient in other languages and could act as translators were available, the study could not recruit older adult gamblers who were only Chinese, Malay or Tamil speaking. It is possible that older adult gamblers who participated in the study would have provided richer descriptions of their gambling activities and cognitive distortions if they had been speaking in their native language. The study recruited only two Malay gamblers, this may be both due to the fact that fewer Malays gamble but it could also be that fewer Malays were willing to declare their gambling status and participate in a gambling study considering the religious and social constraints regarding gambling. The respondents comprised those who wagered money in mahjong, lottery and horse racing—very few were regular casino gamblers and none were regular sports gamblers. Thus, more research is needed to better understand cognitive distortions in these groups. None of the gamblers spontaneously alluded to recognizing these beliefs as distortions either in themselves or in others however, they were not probed regarding their perception of the role played by cognitive distortions in the evolution of gambling

In conclusion, our study is one of the first that has described cognitive distortions among older adults from a multi-ethnic Asian sample. The basic themes of perception of gambling as a skill, near miss, concept of luck, superstitious beliefs, entrapment, gambler’s fallacy, chasing wins, chasing losses, and beliefs that wins are more than losses, played a role in the maintenance and escalation of gambling. Reduction of gambling cognitive distortions and better performance on decision-making were identified as the best predictors of recovery by Rossini-Dib et al. [51] in their study on gambling recovery. Thus, understanding and correcting of these distortions remains a viable approach for the clinical treatment of disordered gambling. Future research must include longitudinal studies to understand causal relationships between cognitive distortions and gambling as well as the role of culture-specific distortions both in the maintenance and treatment of the disorder.

## Supporting information

S1 File Guide for in-depth interviewing. (DOCX)
